# The enigmatic occipital emissary vein, foramina, and canals: anatomical study with application to skull base surgery

**DOI:** 10.1007/s10143-026-04340-8

**Published:** 2026-06-08

**Authors:** Alexandra Campbell, Brianna Hines, Johnathan M. Baudoin, Noritaka Komune, Carmine Antonio Donofrio, Filippo Badaloni, Antonio Fioravanti, Joe Iwanaga, Rizwan Aslam, Kendrick Johnson, Joseph Lockwood, Aaron S. Dumont, R. Shane Tubbs

**Affiliations:** 1 Tulane University School of Medicine, New Orleans, LA USA; 2 Department of Neurosurgery, Tulane University School of Medicine, New Orleans, LA USA; 3 Department of Otorhinolaryngology, Graduate School of Medical Sciences, Kyushu University, Fukuoka, 812-8582 Japan; 4 Department of Neurosurgery, ASST Cremona, Cremona, Italy; 5 Department of Neurosurgery, IRCCS Istituto Delle Scienze Neurologiche di Bologna, Bologna, Italy; 6 Department of Neurology, Tulane University School of Medicine, New Orleans, LA USA; 7 Department of Structural and Cellular Biology, Tulane University School of Medicine, New Orleans, LA USA; 8 Department of Neurosurgery and Ochsner Neuroscience Institute, Ochsner Health System, New Orleans, LA USA; 9 Department of Otolaryngology, Tulane University School of Medicine, New Orleans, LA USA; 10 Department of Anatomical Sciences, St George’s University, St. George’s, Grenada; 11 Department of Neurosurgery, Tulane Center for Clinical Neurosciences, Tulane University School of Medicine, 131 S. Robertson St. Suite 1300, New Orleans, LA 70112 USA

**Keywords:** Skull, Venous, Posterior cranial fossa, Anatomy, Diploic space, Diploe, Skull base, Surgery

## Abstract

Compared with mastoid emissary veins, occipital emissary veins have been less studied, and published descriptions of their foramina are inconsistent. This anatomical investigation aimed to clarify their prevalence, morphology, and relevance to skull base surgery. Two hundred fifty adult skulls were examined for internal and external occipital foramina. Selected samples were sagittally sectioned through the foramina. Ten latex-injected adult cadaveric heads were dissected, then sagittally cut to visualize intradiploic and intracranial venous courses. Portions of vein and surrounding bone were sent for histology. Four additional dry skulls underwent blue latex injection via internal or external foramina followed by selective removal of the inner or outer table. No foramina were present in 16% of skulls. External foramina were absent in 34% and internal foramina in 22%. External foramina connecting to an internal foramen occurred on the left in 10% and right in 8%, typically forming a diagonal pathway with a descending trajectory. Most external foramina were 1–2 cm from midline; 8% were bilateral. Internal foramina clustered near the internal occipital protuberance. Isolated foramina frequently ended in blind pits. Latex-injected specimens demonstrated internal or external veins in 50% of cases, with two showing an accompanying artery. Injection studies revealed no through-passage but did show diploic spread. External openings of the occipital foramina are unreliable surgical landmarks, as their position does not predict intracranial venous entry. Internal emissary veins may exist without an external foramen and risk injury during dural elevation. Separate internal and external diploic communication systems likely exist.

## Introduction

The emissary veins include the condylar, mastoid, parietal, ophthalmic, and occipital veins. Their names and the foramina they traverse are derived from their anatomical positions in the cranium [18]. The lack of valves in many of these veins allows for the regulation of intracranial pressure [11, 20, 37]. The emissary veins also provide alternative venous drainage routes in pathological conditions, which can compensate for hypoplasia of the intracranial venous sinuses. They are also significant in sinus pericranii, which is usually considered a venous malformation but could better be viewed as an adaptive drainage route [29].

The occipital emissary vein (vein of Sperino) is inconsistently present but commonly joins the transverse sinus, torcular Herophili, or occipital sinus [2, 20, 22]. Some have stated that the occipital emissary vein emerges from the skull through the same-named foramen, within the squamous portion of the occipital bone between the external occipital protuberance (inion) (EOP) and the foramen magnum [2]. Externally, it usually joins an occipital vein tributary to enter the suboccipital venous plexus [10, 23]. The occipital emissary veins and their foramina have clinical and surgical significance. However, relatively few studies have evaluated both, and most studies of the foramina have focused solely on the external openings in bony skull specimens. Therefore, the present anatomical study was performed to better understand the anatomy of the occipital emissary vein and its foramina.

## Materials and methods

Two hundred fifty adult skulls were examined for occipital foramina. One hundred fifty were from the Hamann–Todd skeletal collection in the Cleveland Museum of Natural History, Cleveland, OH, USA, and the remainder were from the teaching collection at Tulane University School of Medicine, New Orleans, LA, USA. Ethnicity, sex, and age were known in the 150 skulls from the Hamann–Todd collection. The others had unknown ethnicity, sex, and age, except that they were all adults, as determined by size, dentition, and suture fusion. The age range for the first cohort was 18 to 95 years (mean 55 years; 81 males and 69 females). Lastly, occipital foramina were observed in five fetal skulls with an average age of seven months. The sex of these specimens was unknown.

Externally and internally, the occipital foramina were defined as greater than 0.25 mm in diameter. Microcalipers (Mitutoyo, Japan) and graduated microneedles (0.3 mm to 2 mm diameter) were used for all measurements. External foramina, although located just lateral to the occipitomastoid suture, which drained into the mastoid emissary canal, were not included in our definition of an occipital foramen. Random samples were cut sagittally in the plane of the various occipital foramina.

Additionally, ten latex-injected (blue) adult cadaveric heads with a mean age of 72 years (range 69 to 94 years; five males and five females) were dissected with special attention to the location of the occipital emissary veins. These were sagittally cut with a bone saw to visualize the intradiploic and intracranial courses of the occipital emissary veins further. Samples of the occipital emissary veins and adjacent occipital bone were sent for histological analysis.

Finally, four adult dry skulls underwent instillation of blue latex via manual injection into an internal occipital foramen (*n* = 2) and an external occipital foramen (*n* = 2). The regional inner table or outer table was then drilled away, respectively.

Statistical analysis was performed using GraphPad Prism 10 (Dotmatics, San Diego, CA, USA), with a statistical significance threshold set at *p* < 0.05.

All recently and internationally agreed cadaveric donor study guidelines were followed for the present study [13–16].

## Results

### Gross anatomical findings

**Fig. 1:**
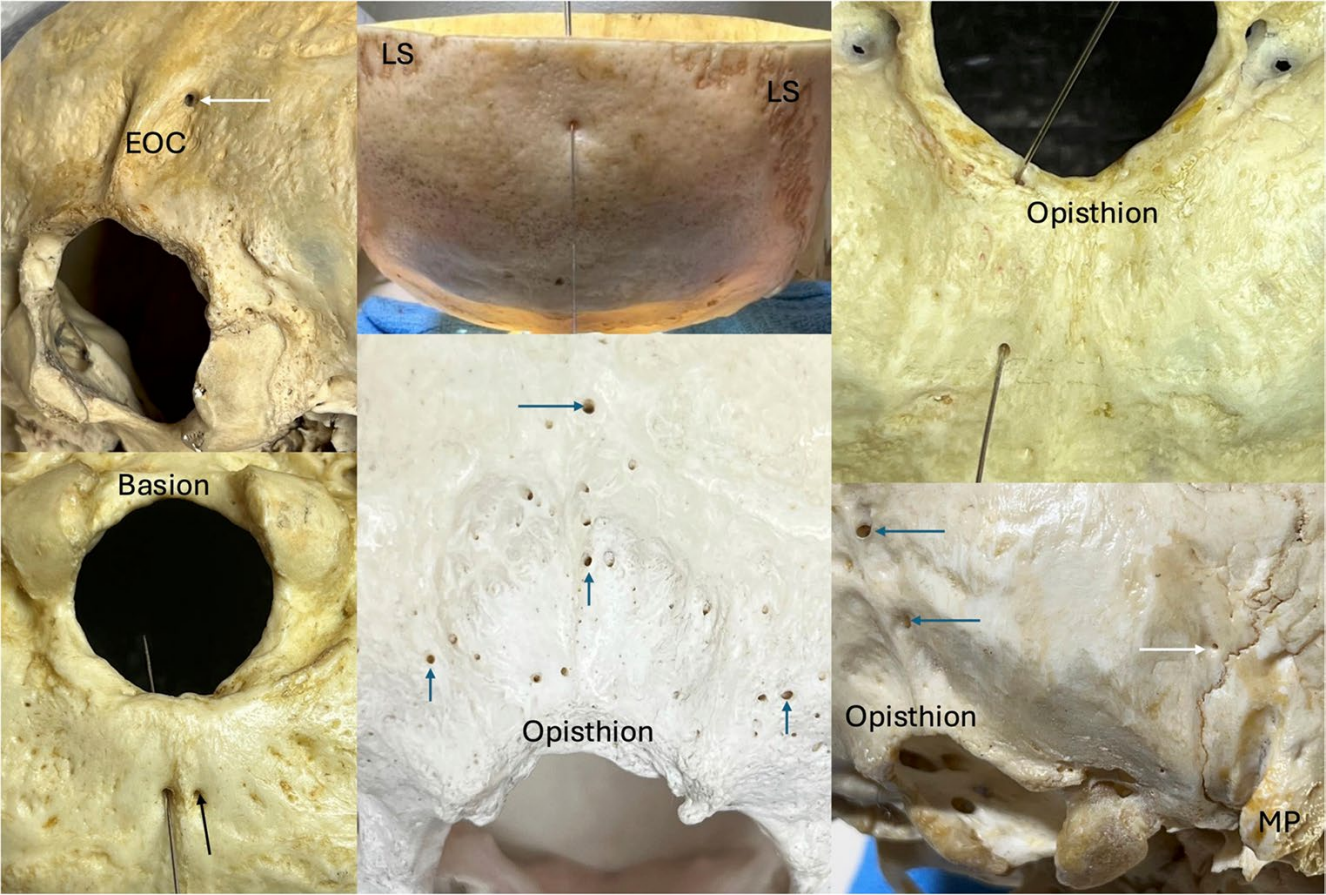
Six examples of external occipital foramina in dry adult skulls. Upper left, large foramen (arrow) to the right of the external occipital crest (EOC). Upper middle, midline external occipital foramen in the interparietal part of the squamous part of the occipital bone. The microneedle is shown entering this foramen and entering the skull via an internal occipital foramen that is at a more superior level. LS=lambdoid sutures. Upper right, right-sided occipital canal with pins placed in the openings. Lower left, bilateral external occipital openings (arrow pointing to left opening) with a microneedle placed through the entirety and entering the intracranium on the right side. Lower middle, note the multiple occipital foramina from an external view, with arrows indicating many of these. Lower right, two occipital foramina (black arrows) to the right of the midline. Also note the small foramen (white arrow) that, although just medial to the occipitomastoid suture and in the occipital bone, drained into the mastoid emissary vein canal and was thus not included as an occipital foramen in our study. MP=mastoid process

**Fig. 2:**
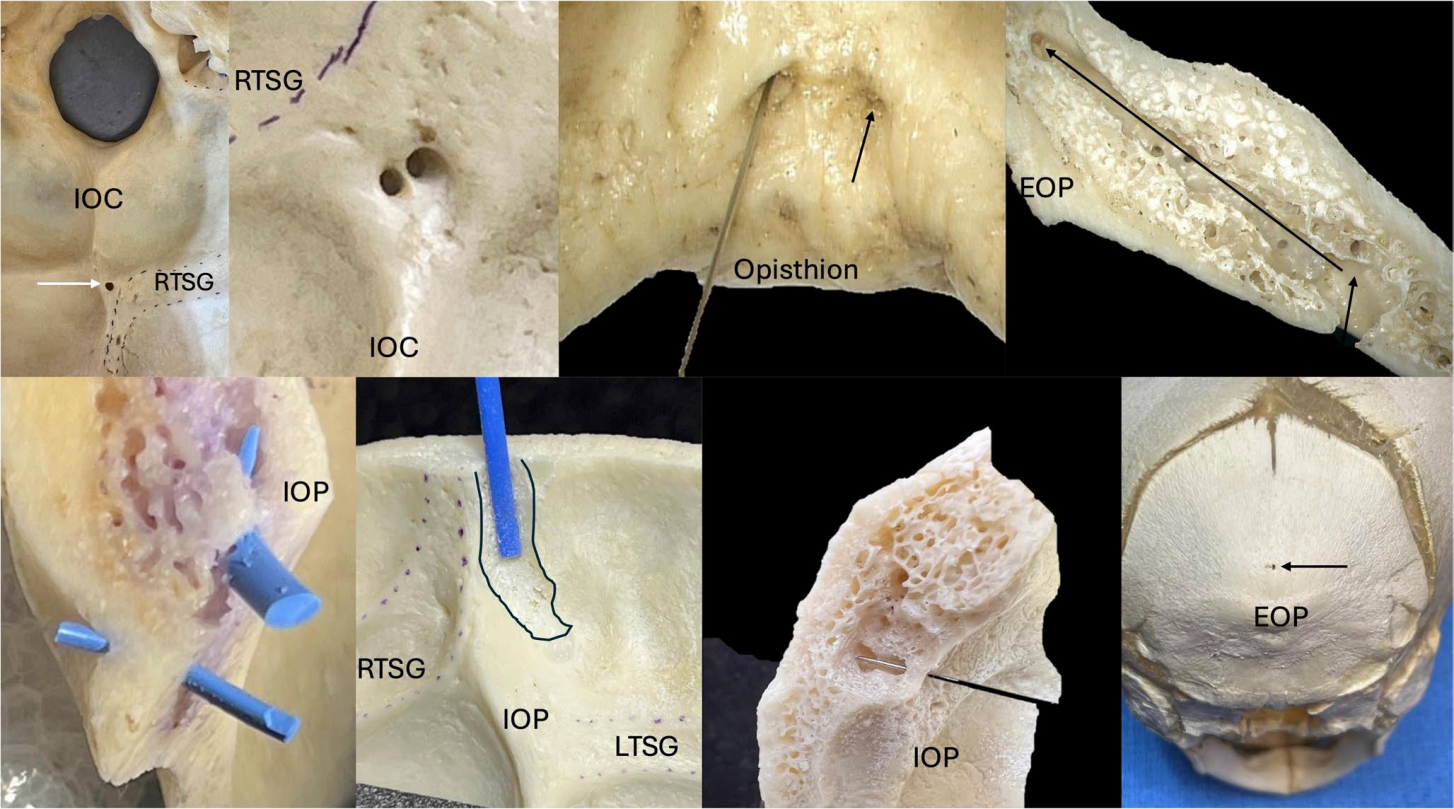
Upper image 1 shows a large internal occipital foramen (arrow) at the internal occipital protuberance superior to the internal occipital crest (IOC), which is near the groove for the right transverse sinus (RTSG). Upper image 2 shows two internal occipital foramina separated only by a bony septum. Note the proximity to the internal occipital crest (IOC) and groove for the right transverse sinus (RTSG). Upper image 3, Two internal occipital foramina (microneedle and arrow) on either side of the midline, very near the posterior foramen magnum. Upper image 4, sagittal section through the occipital bone, noting an external occipital foramen (short arrow) entering into a long, blind ending, occipital canal and the surrounding diploic space. EOP, external occipital protuberance. Lower image 1, sagittal section through the occipital bone near the internal occipital protuberance (IOP) and with blue plastic inserted through an occipital canal that unites the internal and external occipital foramina (lower) and the internal occipital canal that ends in the diploic space (upper). In lower image 2, the occipital canal (outline) has had its adjacent inner table drilled away, and blue tubing has been inserted into it. Note the internal occipital protuberance (IOP), and grooves for the right and left transverse sinuses (RTSG, LTSG). Lower image 3, sagittal section of the occipital bone, noting the internal occipital protuberance (IOP) and microneedle entering an internal occipital foramen and ending in a blind ended cul de sac. Lower image 4 posterior view of an occipital foramen (arrow) in a fetal skull. EOP=external occipital protuberance

Of the 250 adult skulls, no internal or external occipital foramina were identified in 40/250 (16%) skulls. No external occipital foramen was identified in 85/250 (34%), and no internal foramen was identified in 55/250 (22%). External occipital foramina that connected to an internal foramen (as demonstrated with the insertion of a microneedle) were found on the left side in 25/250 (10%) skulls and 20/250 (8%) on the right side of skulls (Fig. 1). The pathway for all of these was diagonal in the majority of skulls and from inside to outside, along a descending trajectory (Fig. 1). One (20%) of five fetal skulls was found to have a single occipital emissary foramen that connected externally to internally (Fig. 2).

The majority of these foramina were located in the supraocciput. In two adult specimens and in the single fetal specimen, the foramen was situated in the interparietal part of the occipital bone (Figs. 1 and 2). For external foramina, one to two were found within one to two centimeters of the midline, with 35 on the left side and 50 on the right side (Fig. 1). Twenty skulls (8%) had bilateral external foramina (Figs. 1 and 2). Ten external foramina were at or near the posterior aspect of the foramen magnum (Fig. 1). The number of midline external foramina was 40 (Fig. 1).

For internal foramina, ten were at or near the posterior aspect of the foramen magnum (Fig. 2). One foramen was identified in the right groove of the oblique occipital sinus, and five were identified in the right groove of the transverse sinus just off the midline. Twenty were in the left transverse sinus groove just off the midline. The majority (345 separate foramina from 210 skulls) of internal occipital foramina were clustered on and near the internal occipital protuberance (Fig. 2). Seventy-one internal foramina were 1 to 2 cm from the internal occipital protuberance. Five of these were found 1 to 2 cm superior to the internal occipital protuberance, and three were within the superior sagittal sinus groove.

Of the external and internal foramina that did not connect with each other, several of them were verified, with sectioning/drilling, to terminate in a blind pit or canal (Fig. 2). Some of these emissary vein canals were lined with cortical bone, and others with dense trabecular bone. Most of the blind-ending canals terminated a short distance into the diploic space of the occipital bone (Fig. 2). However, others extended a longer distance with multiple tributaries extending from the canal into the adjacent diploic space (Fig. 2). Unlike the continuous pathway between some internal and external foramina, canals extending from the internal aspect of the occiput ascended in all specimens with these structures. Table 1 notes the major osteological findings.

**Table 1:** Distribution of external and internal occipital foramina in 250 adult skulls

Category	Finding	Number (*n*)	Percent (%)
Overall skull findings	No internal or external occipital foramina	40	16%
Overall skull findings	No external occipital foramen	85	34%
Overall skull findings	No internal occipital foramen	55	22%
External occipital foramina	External foramina connected to internal foramina (left side)	25	10%
External occipital foramina	External foramina connected to internal foramina (right side)	20	8%
External occipital foramina	Bilateral external foramina	20	8%

**Fig. 3:**
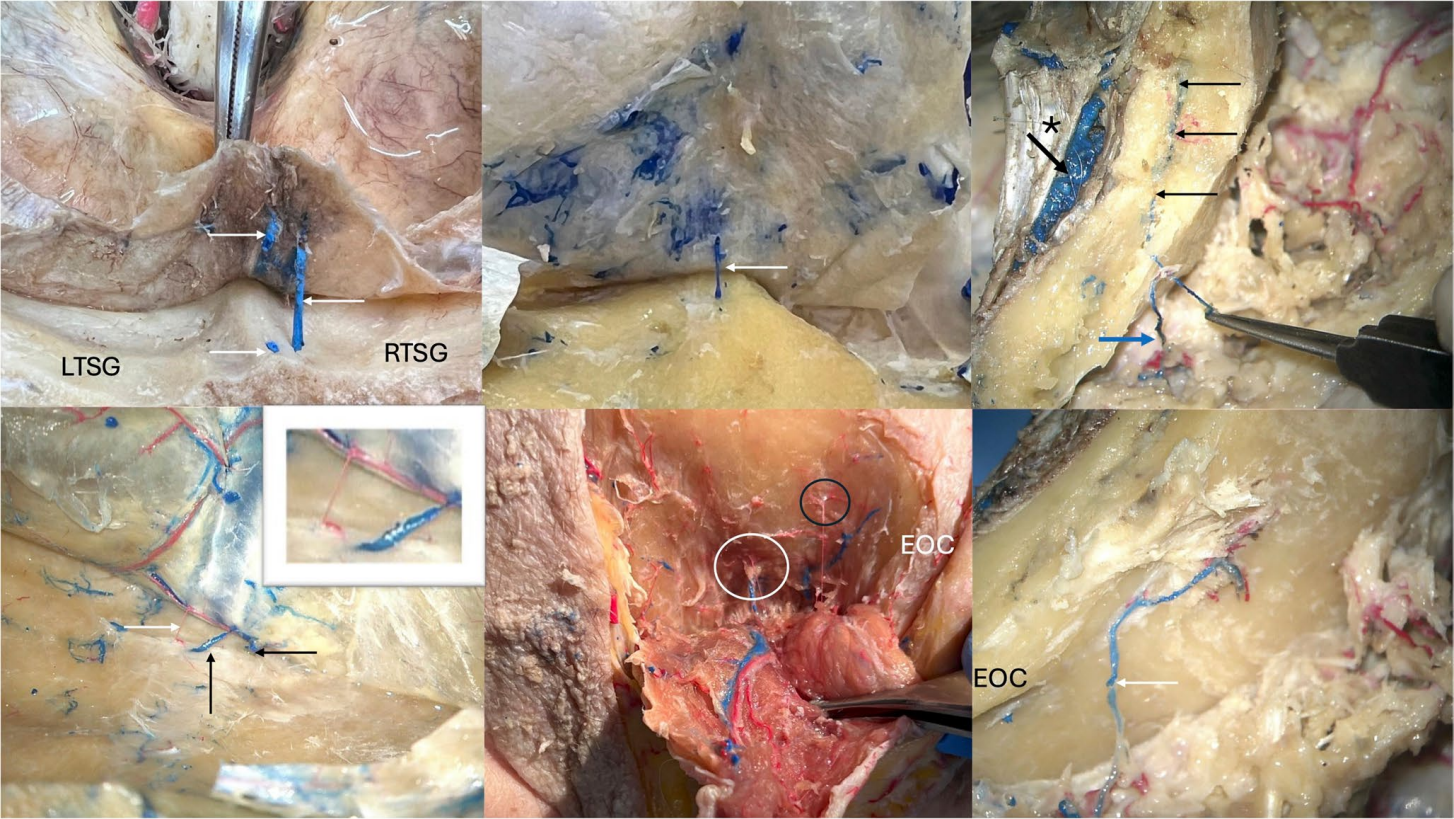
Latex-injected cadaveric specimens. Upper left. Following retraction of the dura mater covering the internal surface of the occipital bone, two veins (arrows) are shown connecting the area of the torcular Herophili to the occipital diploic space. The left vein was cut, and there were no external occipital foramina. The grooves for the left and right transverse sinuses are shown (LTSG, RTSG). Upper middle. Internal view following retraction of the dura mater covering the internal surface of the occipital bone with a single vein (arrow) joining the torcular to the diploic space of the occipital bone at the internal occipital protuberance. Upper right, sagittal section in the midline, noting external veins (blue arrow and forceps) entering the occipital bone, ascending within an occipital canal (three small black arrows), and draining internally near the torcular. For reference, the falx cerebelli (*) and occipital sinus (large black arrow) are shown. Lower left. Following retraction of the overlying dura mater, small veins (black arrows) and arteries (white arrow) are shown connecting the left transverse sinus and meningeal branches of the occipital artery to the internal aspect of the occipital bone. This anatomy is magnified in the inset. Lower middle. External view of veins (white circle) and arteries (black circle) entering the occipital lateral to the external occipital crest (EOC), into the left surface of the occipital bone following retraction of the overlying suboccipital musculature. Bottom right. Occipital vein (white arrow) entering the occipital bone lateral to the external occipital protuberance (EOC)

Of the latex-injected cadaveric specimens, five (50%) were found to have either an internal occipital emissary vein or an external emissary vein (Fig. 3). Two specimens, in addition to an occipital emissary vein, were also found to have an artery traveling into an occipital foramen (one into an external foramen and one into an internal foramen) (Fig. 3).

**Fig. 4:**
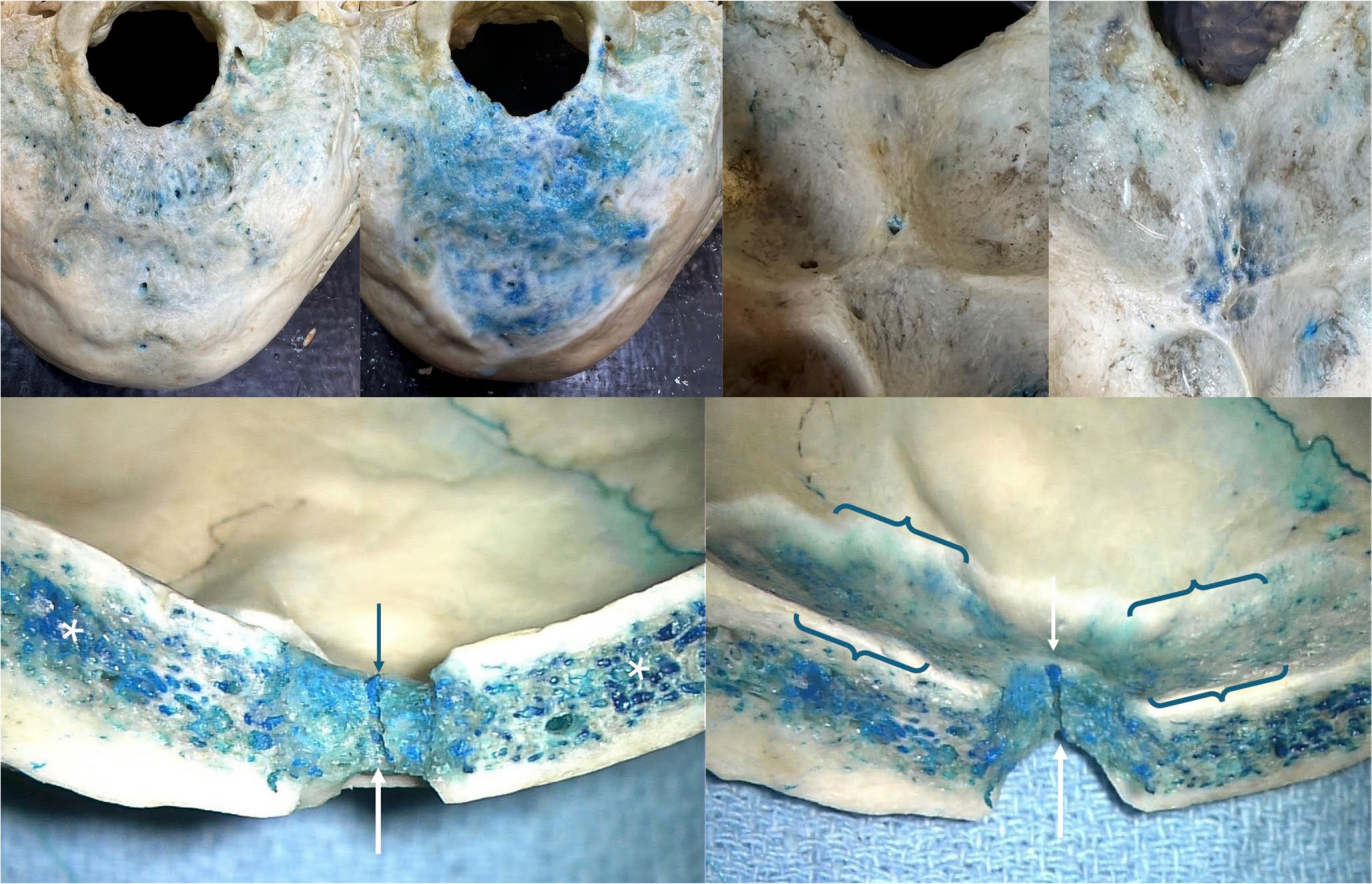
Upper image 1 shows the external occipital bone following injection of blue latex into an external occipital foramen. Note the blueish color in the diploic space. Upper image 2 shows upper image 1 after drilling away the outer table of the occipital bone. Note the spread of blue latex. Upper image 3 shows the internal occipital bone at the internal occipital protuberance following injection of blue latex into an external occipital foramen. Upper image 4 shows the spread of the injected latex in upper image 3 after drilling away the inner table of this region, withminimal spread of latex into the region’s diploic space noted. Lower images show a small occipital canal (arrows) and spread of latex into the superior and inferior diploic space (*) in a sagittal view (left) and posteromedial view (right) with spread into the right lateral diploic space (parentheses)

For the four dry skulls that underwent latex injection, none had latex egress from an occipital foramen externally if injected internally or internally if injected externally. Injection into an external foramen resulted in a wide spread of latex into most of the supraocciput diploic space (Fig. 4). However, injection into an internal foramen had minimal spread (Fig. 4).

Although there were slightly more foramina noted on the left sides, this, and any difference between male and female skulls, did not reach a statistically significant difference.

### Histological findings

**Fig. 5:**
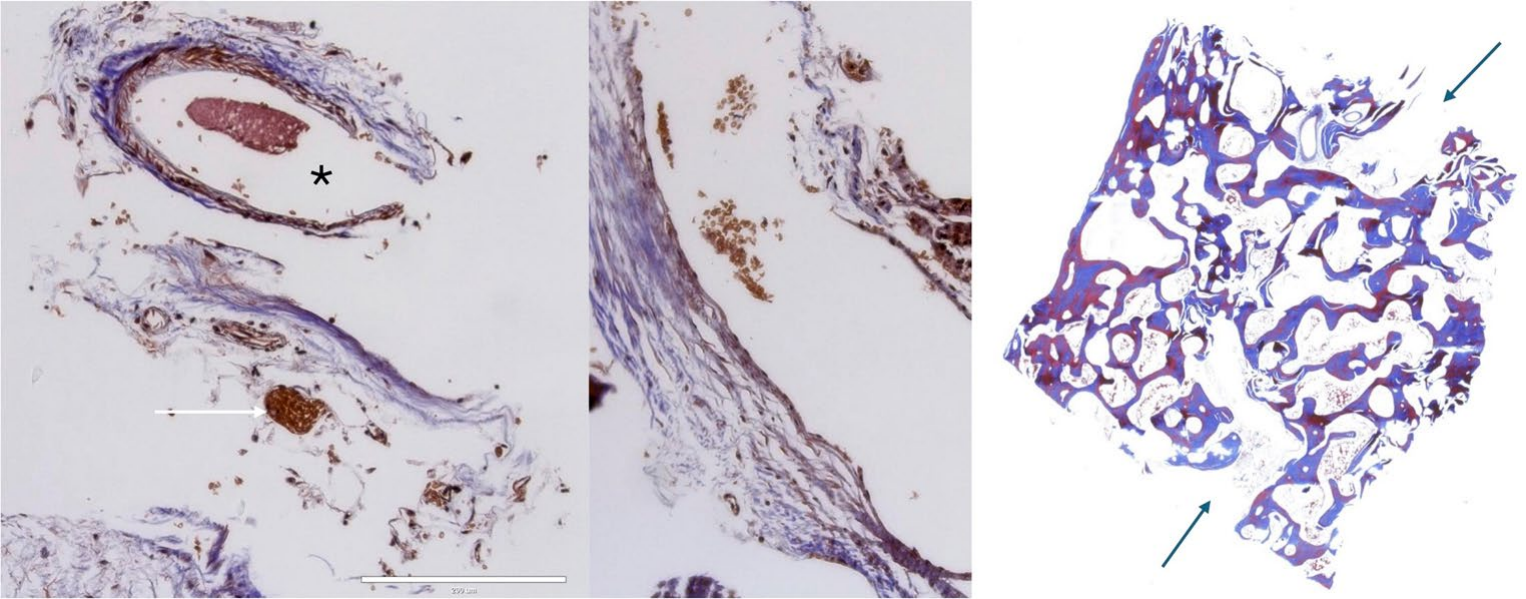
Histological images of the occipital veins. The left axial image shows the vein (*) with blood cells and an adjacent nerve fiber (arrow). The middle image is a longitudinal section through the vein containing blood cells. The right image is a decalcified section through the occipital bone showing the extensive diploic space and entrance of veins into the internal and external surfaces of the bone (arrows)

Histological examination of tissue sections obtained from the external and internal openings of the occipital foramen revealed medium-caliber vessels consistent with veins. The sections were stained with Masson’s trichrome (MT) and examined under light microscopy at 20× and 40× magnification. The vessel walls displayed well-defined layers of collagen and smooth muscle with a dense, blue-staining tunica externa surrounding more lightly stained smooth muscle fibers of the tunica media (Fig. 5). The lumina of many vessels contained blood cells.

In both cross-sectional and longitudinal planes, there were numerous dark brown- to reddish-brown-staining nerve fibers, closely associated with the external aspect of the vessel wall (Fig. 5). These fibers appeared in small, irregular bundles and occasionally as single strands. Their distribution was concentrated along the adventitia of the vessel, and they were embedded within the surrounding collagenous connective tissue. The absence of perineurial sheaths, fascicular organization, or evidence of thickly myelinated fibers suggested they were autonomic rather than somatic nerves. No large-caliber somatic motor or sensory nerves were seen in the plane of section.

The most notable finding was of perivascular nerve fibers closely associated with the outer wall of the emissary vein. These appeared as brown-stained bundles and individual axons. Given their size, organization, and perivascular orientation, they are best classified as sympathetic postganglionic autonomic fibers. Decalcified specimens with emissary veins entering the occipital bone could not be shown in continuity through the occipital bone. Rather, the external vessel entered the diploic space and then lost its identity, just as the internal vessels.

## Discussion

We identified that 40 out of 250 (16%) individuals had neither an internal nor an external occipital foramen. No external occipital foramen was identified in 85/250 (34%), and no internal foramen was identified in 55/250 (22%). External occipital foramina that connected to an internal foramen (as demonstrated with the insertion of a microneedle) were found on the left side in 25/250 (10%) skulls and 20/250 (8%) on the right side. Connections from intracranial venous sinuses to the diploic space of the occipital bone with no external occipital foramen can be interpreted as direct drainage of the sinuses into this space, as recently reported for the basilar venous plexus [38] and hypothesized for the occipital emissary vein in some cases [17].

### Occipital emissary veins

Embryologically, most emissary veins, such as the occipital emissary vein, are distinguishable at 3.5 months of gestation [23]. The evolutionary explanation for the development of the occipital emissary veins, and of emissary veins overall, could relate to the selection for bipedalism in *Homo sapiens* [8]. These vessels permit the delivery of venous blood to the vertebral venous plexus, which is necessary when standing erect; this position diverts most of the cerebral venous outflow from the internal jugular veins to the vertebral venous system [28].

These physiological characteristics have practical implications in certain pathological conditions, such as pseudotumor cerebri [12] and craniosynostosis [31], as well as in the selection of surgical positioning. For example, elevated intracranial pressure can augment venous outflow through the vertebral venous plexus, a phenomenon that may be further accentuated in the semisitting position [7]. Occipital emissary vein injuries can result in massive intraoperative bleeding [31]. Such hemorrhages can be so severe that some authors have recommended preoperative MRI for such pathologies [11].

To exemplify the significance of enlarged occipital emissary veins, Thompson et al., reported a case of cranial vault remodeling in a patient with kleeblattschädel deformity. After the occipital emissary veins were divided, the resultant intracranial hypertension resulted in the death of the patient. Postmortem examination revealed that intraosseous veins had resulted in the formation of a collateral system that was responsible for draining most of the intracranial venous blood; these occipital emissary veins were visible on retrospective review of the preoperative MRI [34].

Investigators have proposed a role for the occipital emissary veins in the pathophysiology of headache in patients whose occipital vein drains into the deep cervical veins. Contraction of the muscles surrounding the deep cervical veins could obstruct flow and increase intracranial pressure [26]. Dilated emissary veins have also been reported as an unusual cause of tinnitus [5]. Because they are valveless, these veins are a potential source of infection traveling intracranially from superficial tissues [1].

Some have suggested that the occipital emissary veins can serve as surgical landmarks for localizing the intracranial dural venous sinuses during operations on the posterior cranial fossa [1, 9]. However, based on our findings, the veins and foramina are too variable to serve as reliable intraoperative landmarks. Additionally, thrombosis of these veins during interventional procedures or during surgery could result in concomitant intracranial venous sinus occlusion. However, based on our anatomical findings, a direct, horizontal pathway from external to internal is very uncommon. Lastly, if abnormally enlarged, these veins might be surgical obstacles and could result in significant hemorrhage [27].

Additionally, some have mentioned confusion in the literature’s descriptions of the occipital vein, occipital emissary vein, and occipital diploic vein [17]. An emissary vein, by definition, should unite an intracranial venous sinus with an extracranial vein [4, 7]. Along this connection, tributaries to the diploic space occur. However, based on our study, this occurs with the occipital emissary vein uncommonly. Moreover, the occipital veins have been found to most commonly drain into the mastoid emissary vein [18]. Therefore, we would suggest that the occipital emissary vein, per se, does not exist, and in most cases, two sets of veins can exist, singularly or together. One set drains the veins of the external occipital region to the occipital diploic space, and the other drains the intracranial venous sinuses to this same space (Figs. 3, 4 and 5).

### Occipital emissary foramina

Most previous studies have examined the occipital foramina from an external perspective. In the present study, no internal or external occipital foramina were observed in 16% of specimens. An external occipital foramen was absent in 85 out of 250 cases (34%), while an internal foramen was absent in 55 out of 250 (22%). External occipital foramina communicating with an internal foramen were identified in 10% of skulls on the left side and in 8% on the right. The occipital emissary foramen is located on the squamous part of the occipital bone [9, 21, 32, 33].

An MR study has reported the incidence of the occipital emissary vein as 28.6% [11], but the prevalence of the foramen varies widely among cadaveric studies. Several authors have reported a prevalence of less than 3%, ranging from 0.47% to 2.7% [3, 9, 25, 30]. Other studies, however, have documented higher rates: 9.5% reported by Singhal and Ravindranath [33], 14.1% by Murlimanju et al., [21], and 36.2% by Singh [32].

Although the occipital emissary foramen is most commonly single, bilateral foramina have also been reported; in one study, they accounted for 14.28% of skulls presenting occipital emissary foramina [33].

We found that the location of the external opening of the occipital foramen varies slightly, correlating with the dural venous sinus into which the vein drains [9, 21, 32, 33]. It is most frequently reported on the left side, although right-sided or midline positions also occur [9]. In some cases, it is located on or adjacent to the external occipital crest or the EOP (Fig. 3; [32]).

**Fig. 6:**
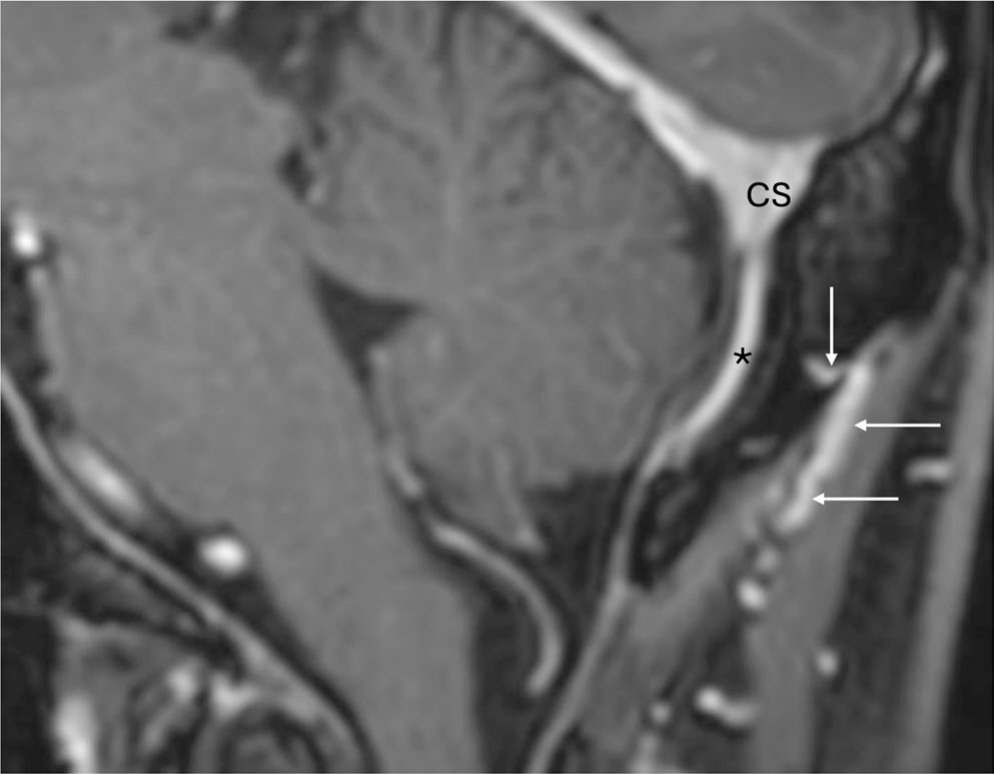
Sagittal contrasted MRI noting an occipital vein (two horizontal arrows), emissary vein (vertical arrow) entering the diploic space, and occipital sinus (*) and confluence of sinuses (CS) (torcular Herophili)

Although several authors have emphasized the importance of identifying the occipital emissary foramen/vein and other posterior fossa emissary veins before performing suboccipital craniotomy or posterior cranial fossa surgery, in order to minimize excessive bleeding, our findings do not support this recommendation [24]. The external occipital foramina containing veins often did not correspond to the level of the dural venous sinus into which they drained, and in many cases, the external foramina had no intracranial connections [1, 21]. However, MRI (Fig. 6) and MR venography can help to distinguish a large occipital emissary foramen from a giant arachnoid granulation or an osteolytic lesion, both of which it may mimic on CT [39].

**Fig. 7:**
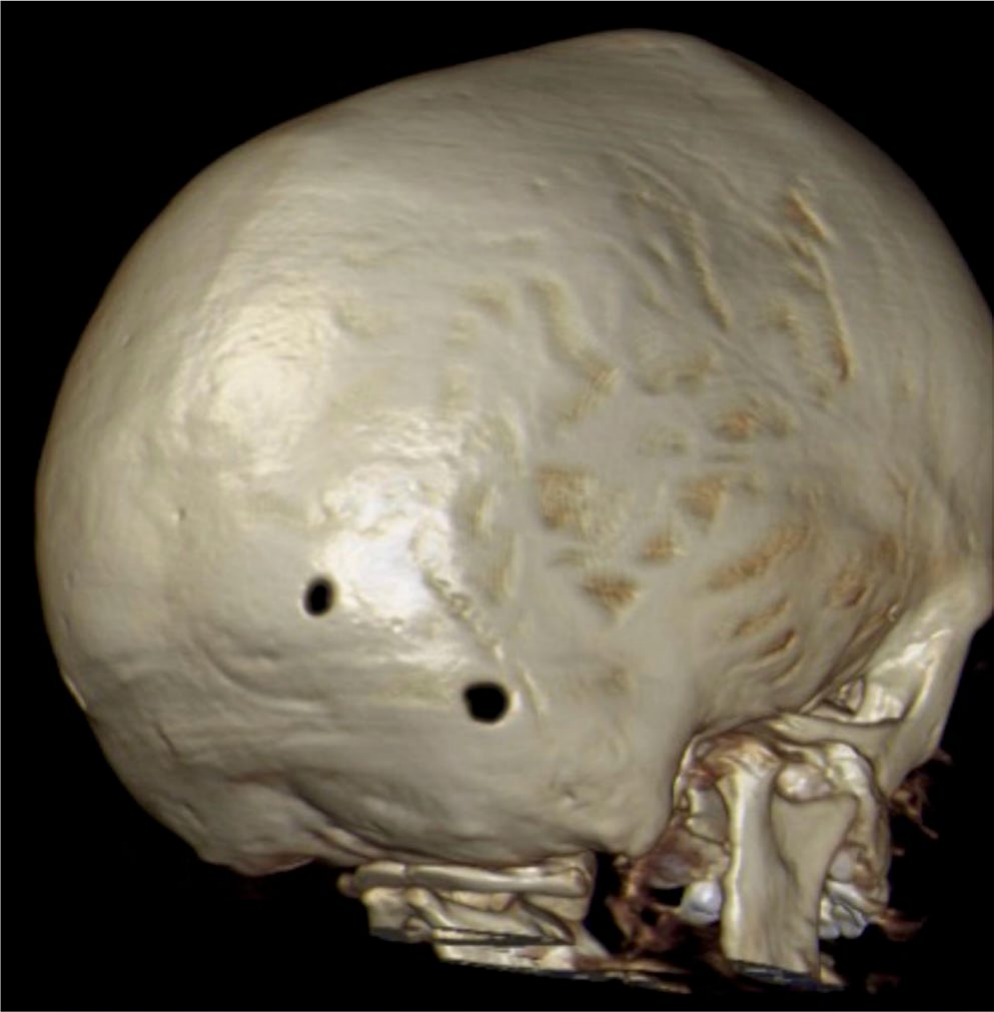
3D CT reconstruction of a child with pansynostosis and found to have giant foramina located in the occipital bone, which indicate larger than normal occipital emissary veins traversing the occiput and a potential hazard with any cranial surgery in this region

In a study including 1500 skulls, Boyd reported a large (10 mm) occipital emissary foramen associated with oxycephaly in two cases [3]. In our clinical experience, such foramina in patients with pansynostosis of the calvarial sutures may present as giant occipital foramina (Fig. 7).

### Occipital Emissary Vein Canal

The occipital emissary canals have been described as following tortuous and branching courses [6]. We observed that these canals rarely traverse the entire thickness of the occipital bone; instead, they most often terminate in a blind sac within the diploic space (Figs. 2 and 5). This finding is consistent with previous reports noting that emissary canals often exhibit highly variable morphology and obscured openings, making them difficult to identify on MRI [35].

On imaging, a dilated occipital emissary vein canal may mimic a bony defect caused by an arachnoid granulation, sinus pericranii, meningoencephalocele, or dermoid cyst [6]. Enlarged foramina or canals can also be indicative of increased intracranial pressure [6]. Additionally, emissary canals may become dilated in the presence of a hypoplastic internal jugular vein or a vascular malformation [1, 19, 36, 38].

## Conclusions

The occipital emissary vein, foramen, and canal have received limited attention in the literature, and their incidence and course remain ambiguously defined. The external opening of the occipital emissary vein foramen generally does not correlate with the plane at which the vein enters an intracranial dural venous sinus, which is an uncommon arrangement in itself, rendering these external openings unreliable as surgical landmarks. Our findings also indicate that, even in the absence of an external occipital emissary vein foramen, internal veins may be present and can be injured during mobilization of the dura mater from the internal surface of the occipital bone. To our knowledge, this is the first study to document arteries and nerves traversing some occipital foramina.

In most cases, there are two different venous systems communicating with the occipital diploic space: an internal system connecting the diploic space to the dural venous sinuses, and an external system linking the veins of the external occiput, such as the occipital vein, to the diploic space. Therefore, an occipital emissary vein in the classical sense is rarely encountered.

A comprehensive understanding of the occipital emissary vein and its variations may provide significant clinical benefits, particularly in neuroimaging interpretation and skull base surgery, having practical implications in cases of pathologically increased intracranial pressure or in the selection of surgical positioning, both of which can augment venous outflow through the vertebral venous plexus.

## Data Availability

No datasets were generated or analysed during the current study.

## Declarations

The authors have nothing to disclose.
